# Investigation on the role of surfactants in bubble-algae interaction in flotation harvesting of *Chlorella vulgaris*

**DOI:** 10.1038/s41598-018-21629-x

**Published:** 2018-02-19

**Authors:** Zhou Shen, Yanpeng Li, Hao Wen, Xiangying Ren, Jun Liu, Liwei Yang

**Affiliations:** 1 Key Laboratory of Subsurface Hydrology and Ecology in Arid Areas, Chang’an University, Xi’an, 710054 China; 2 School of Environmental Science and Engineering, Chang’an University, Xi’an, 710054 China; 3 School of Civil Engineering, Chang’an University, Xi’an, 710061 China

## Abstract

In this work, a fundamental study was carried out on the role of surfactants in bubble-algae interaction to improve the understanding of how surfactants influence the flotation performance. Flotation tests for harvesting *Chlorella vulgaris* were first conducted using two surfactants, hexadecyltrimethyl ammonium bromide (C_16_TAB) and tea saponin. The effect of surfactants on harvesting efficiency was found to depend on their type and concentration. The present results also indicated that C_16_TAB exhibited higher harvesting efficiency than tea saponin. The adsorption experiments of surfactants onto *C*. *vulgaris* and the characterization measurements of algae surface were then carried out to reveal underlying interaction mechanisms between surfactants and algae in air flotation process. The results confirmed the adsorption process of surfactants onto *C. vulgaris* was feasible, spontaneous and endothermic. Subsequently, two mechanism models were proposed to qualitatively establish the interaction relationship among algae, surfactants and bubbles in the flotation. According to two models, C_16_TAB could neutralize the algal potential, while tea saponin converted algal surface from hydrophilic into hydrophobic. Overall, two surfactants used here could facilitate attachment of *C. vulgaris* onto bubbles, making the algae easier to be harvested, thereby increasing the flotation recovery.

## Introduction

Algae are regarded as a promising resource because it is renewable and environmentally friendly; hence, algae-based bioproducts such as biodiesel and health products are becoming more and more concern nowadays. Putting algae and traditional crops like maize into comparison, algae need less land area and thus don’t compete with food crops for space^1^. Besides algae contain a high proportion of fatty acid and lipid^2^. However, the concentration of algae in nutritious solution is too low to meet the standard for downstream process. In order to enrich algae from cultured solution, it is urgent to develop a cost-effective and efficient harvesting method.

Although several methods e.g. filtration^3^, centrifugation^4^ and electrolysis^5,6^ have been applied to algae harvesting, their energy-intensiveness make them inapplicable^7^. On the contrary, flocculation is the low-cost and energy-efficient technique, but it requires a long time for sedimentation to occur which makes it less efficient^8^. Air flotation is widely considered as a promising approach on account of its rapid, easy and effective capture of algal cells from the cultured solution by applying an extra dosage of surfactants^9^. In the algae harvesting by air flotation, the interaction between gas bubbles and algae is critical to the formation of stable bubble-algae aggregates, which is a fundamental step required for achieving higher efficiency in harvesting. Generally, bubble-algae interaction can be divided into three processes: collision, attachment and detachment^10^. With approaching of the bubble and algae to the contact distance, collision can occur. The collision process is determined mainly by the hydrodynamics governing bubble-algae approach in the liquid phase. As the algae and bubble come closer, the influence of intermolecular and interfacial forces is decisive for attachment and detachment processes.

Among these three processes, addition of surfactants has been reported to improve the flotation performance by mainly influencing the attachment step. The usage of surfactants has significant effect on bubble behaviors^11,12^. On the other hand, it can modify surface feature of algae^13^ so that the upcoming tiny bubbles can attach to the algal surface easily and carry algae upward to the top of the solution. Numerous studies on the selection of surfactants have been reported in the application of algal harvesting. For example, Coward *et al*.^14^ employed Ecover for algal harvesting which is a mixture of biodegradable surfactant (15% anionic surfactant, 5% nonionic surfactant) produced from yeasts, glucose, and rapeseed oil, and obtained an optimal concentration of 0.15 mg/L. Garg *et al*.^15^ had employed the tetradecyltrimethylammonium bromide (C_14_TAB) and dodecyl ammonium hydrochloride (DAH), and suggested that C_14_TAB and DAH were advantageous to improve the harvesting efficiency. A cation surfactant hexadecyl trimethyl ammonium bromide (C_16_TAB) has also been reported to be a favorable surfactant for *C. vulgaris* removal^14,16,17^, as it could improve hydrophobicity of the algal surface well^13^. Moreover, tea saponin is a representative biodegradable biosurfactant, and it could also help to harvest the *C. vulgaris* significantly^18^.

However, the interaction mechanisms between surfactants and algae and how the surfactants influence the flotation performance have not been completely understood. Different kinds of surfactants have different adsorbed ratios onto the algae, and adsorption process is supposed to play key role in attachment process. In this way, surfactants could contribute to the harvesting performance. Therefore, a study on the adsorption between surfactants and algae seems a feasible way to explore the above mechanism. Recently, several studies on algal adsorbing heavy metal and magnetic beads have been conducted. For example, Liu *et al*.^19^ studied the adsorption of functional graphene-based magnetic nanocomposites onto *Chlorella*, and demonstrated that the valence forces mainly controlled the overall rate of adsorption process. Moreover, organics may restrain the adsorption process, in which a large number of carboxyl and phenolic hydroxyl groups would react with heavy metals in the solution, thereby reducing the adsorption quantity onto microalgae^20^. In contrast, inorganic salts such as phosphorus may promote the adsorption of Cu onto *Chlorella pyrenoidosa*^21^. However, few works have been conducted into the adsorption between surfactants and algae in flotation process.

The aim of this work is to investigate the role of surfactants in algae harvesting to improve the understanding of how surfactants influence the attachment performance in flotation process. For this purpose, *Chlorella vulgaris* was selected as the experimented strain of algae because it is one of the most common energy microalgae with high oil content (>20%) and strong survival ability, and has been widely employed in algae harvesting process^22–24^. *Chlorella vulgaris* were treated by two kinds of surfactants (C_16_TAB and tea saponin). The adsorption of surfactants was then studied to explore the driving force of the process. After that, comparisons of functional groups and surface characteristics of algae before and after adsorption were carried out to examine whether there was any underlying chemical or physical behavior. Finally, the mechanism models were proposed to qualitatively describe the interaction among algae, surfactants and bubbles in the flotation.

## Materials and Methods

### Cultivation of algae

The microalgal strains (*Chlorella vulgaris*, FACHB-8) used in this study were obtained from Freshwater Algae Culture Collection at the Institute of Hydrobiology (FACHB-collection, China). A photobioreactor (PBR, Shanghai Guangyu Biological Technology Co, Ltd. China) constructed in Pyrex with the base dimensions of 25 cm and a height of 100 cm was used for algae cultivation. *C. vulgaris* was grown at 25 ± 3 °C under a light intensity between 3000 and 3500 Lux (12 h/d) the air flow rate at 15 lit/h. The algae was cultured in BG-11 nutrient medium, with 1.5 g/L NaNO_3_, 0.04 g/L K_2_HPO_4_, MgSO_4_·7H_2_O, and 1 ml/L A5(trace mental solution), etc^25^. The pH was maintained at 7–7.5 by using 1 mol/L NaOH and HCl using a peristaltic pump automatically. During the cultivation of algae *C. vulgaris*, it stayed in start phase for four days, exponential growth phase for 12 days and then went into the stationary phase (less than 5% increase in the cell numbers per day). Microalgae at stationary growth phase were washed twice with distilled water and then used to prepare algal suspension samples for the following experiments and measurements.

### Adsorption procedure

Each time, 100 ml of *C. vulgaris* in a stationary phase with a certain dose of flotation agent was mixed in a conical flask with a cover, which was then shaken in a thermostat shaker at 150 rpm. This study employed C_16_TAB and tea saponin surfactants as adsorbents. Concentration of both the surfactants was measured by UV spectrophotometry (Shimadzu, Japan), where methyl orange^26^ and vanillin-concentrated sulfuric acid were used as indicators for C_16_TAB and tea saponin respectively^27^.

#### Adsorption kinetics

Adsorption kinetics was used to determine whether the adsorption process is physical adsorption or chemical adsorption in this study. The experiments of adsorption kinetic were conducted at a constant temperature of 25 °C (298 K, pH 7.0). The initial concentration of each sample was set as 25 mg/L, and the samples were filtered using membranes of 0.22 μm at predetermined time intervals. Each experiment was duplicated under identical conditions, also for each time interval two replicated samples were considered. Blanks containing no flotation agent were analyzed and the loss (generally quite low) was considered. The uptake of flotation agent at time *t*, *Q*_*t*_ (mg/kg) was then calculated using the following equation:

1 $${Q}_{t}=\frac{V({C}_{0}-{C}_{t})}{m}$$

where *C*_0_ and *C*_*t*_ are the initial concentration of surfactant (mg/L) and concentration at time *t*, respectively, *V* is the volume of the solution (L), and *m* is the weight of the sediment samples (g).

#### Adsorption isotherm

Adsorption isotherm was used to determine whether the adsorption behavior is monolayer or multilayer in this study. The algal solutions with different concentrations (25, 50, 100 and 150 mg/L, at pH 7.0) of the C_16_TAB or tea saponin were used here. The equilibrium time was set according to the results obtained from the kinetic studies, making sure that the time is long enough. The aqueous samples were filtered using the membranes of 0.22 μm. The concentrations of flotation agent were analyzed by UPLC (Ultra Performance Liquid Chromatography). For each of the initial concentration two replicated samples were used. The uptake of flotation agent at equilibrium, *Q*_*e*_ (mg/kg) was then calculated by using the following equation:

2 $${Q}_{e}=\frac{V({C}_{0}-{C}_{e})}{m}$$

where *C*_*e*_ is the equilibrium concentration of surfactant (mg/L) in the solution.

### Flotation experiments

Flotation experiments were carried out using a 1.0 L Denver Flotation Cell (ShunZe, XFD-1, China). The microalgal cultures were first stirred vigorously for 2 min, weighed, and the density of cells was calculated by using the hemocytometer, followed by transferring them into the flotation cell. The pH of flotation pulp was adjusted with HCl (0.1 mol/L) or NaOH (0.1 mol/L) before adding C_16_TAB. Initially, microalgal suspension was conditioned by mixing at 800 rpm for 5 min, then at 600 rpm for 10 min for the flotation test. All the flotation harvests were conducted under an air flow rate of 180 L/h. All the results are presented as the average of three measurements^15^.

Microalgal harvesting efficiency (HE) was determined by using the following equation:

3 $$HE=1-\frac{Tt}{Ff}$$

where *T* is the wet mass of the tailing (or sink left in the flotation cell), *F* is the wet mass of the feed and *t* is the microalgal concentration in the tailing.

### Zeta potential

The zeta potential of microalgae cells in the samples was determined using a Zetasizer (Beckman Coulter, Delsa^TM^Nano, USA). To minimize the effects of settling, the samples were kept undisturbed for 10 min, allowing the flocs to settle and then the cultured algae was used for the measurements. For each sample, Zeta potential measurements were performed for at least three times.

### FT-IR spectroscopy

Spectra were collected using a Fourier transform infrared spectroscopy (FT-IR) spectrometer (Nicolet6700 Thermo Fisher, USA) equipped with a deuterated triglycerine sulphate detector, which uses an HTS-XT high-throughput microplate extension. The spectral scan was made in the range of 4000–400 cm^−1^ with four scans for each sample. For this, 1 mL sample was taken from each flask and subjected to centrifugation at 14000 g for 5 min. The supernatant was discarded and the pelleted wet biomass was weighed. Each sample was then normalized to a concentration of 60 mg/ml with deionized water. Each sample of 30 μL was then pipetted onto 96-well silicon microplate and dried at 40 °C overnight.

## Results and Discussion

### Flotation experiments

**Figure 1:**
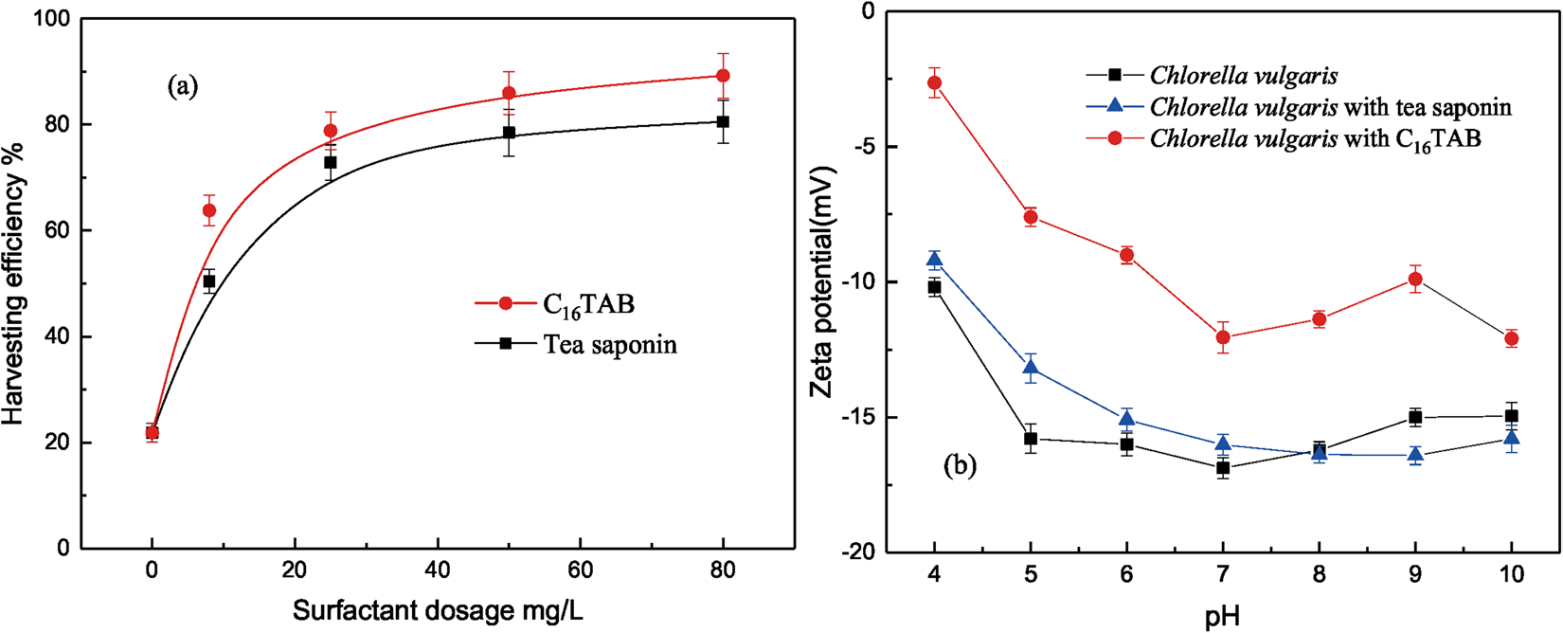
Effect of surfactant dosage on flotation recovery (**a**) and Zeta potential of *Chlorella vulgaris* vs pH in different solutions (**b**).

The effect of concentration of surfactants on harvesting efficiency was presented in Fig. 1. It could be observed that the harvesting efficiency increased rapidly with an increase in the surfactant dosage till 25 mg/L. The maximum flotation recovery was 89.23% for C_16_TAB, whereas 80.53% for tea saponin. This suggests that the adsorption saturation state may be achieved under high concentration of surfactants. Moreover, the addition of C_16_TAB exhibited higher harvesting efficiency than tea saponin. This is similar to the result from Agnes *et al*.^28^, in which C_16_TAB was more effective in harvesting *Scenedesmus obliquus*, in 40, 60 and 80 mg/L. This difference should be mainly attributed to different electrostatic forces existing between algae and surfactants and the amount of hydrophobic part within two surfactants. This could be supported by Zeta potential measurements of *C. vulgaris* obtained from different surfactant solutions as shown in Fig. 1(b). It can be seen that *C. vulgaris* was negatively charged and the addition of C_16_TAB increased the Zeta potential of *C. vulgaris* because of its positive charge, whereas adding neutral tea saponin did not influence the Zeta potential apparently. The changes of Zeta potential after adding two surfactants respectively were also proven by two previous studies^13,28^. Furthermore, the harvesting efficiency of flotation process can be affected by the electric charge of the bubble. Kwak and Kim^29^ found that bubbles constituted by either air or carbon dioxide carried the negative charge in pH range of 4 to 11. Therefore, the algae can connect bubbles with C_16_TAB by electric force.

### Adsorption kinetics

**Figure 2:**
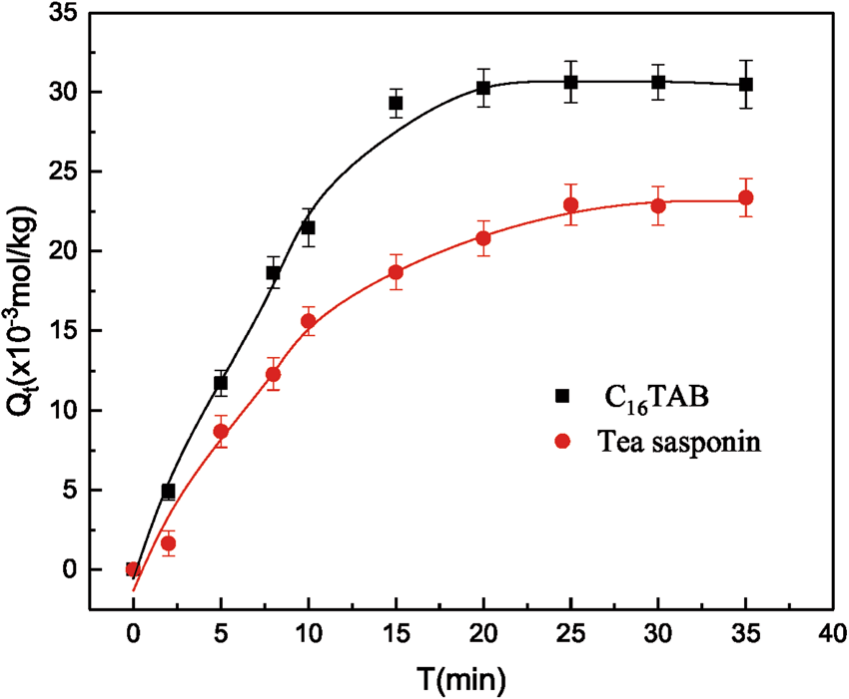
Effect of contact time on the adsorption of C_16_TAB and tea saponin.

The adsorption of C_16_TAB and tea saponin onto *C. vulgaris* as a function of contact time at the natural pH (~7.0) was shown in Fig. 2. The results from this figure elucidated that the amount of adsorption of both surfactants increased gradually with an increase in the treatment time until adsorption equilibrium was reached. Adsorption equilibrium time was about 20 min for C_16_TAB and 25 min for tea saponin, respectively. The equilibrium time for two surfactants was much shorter than that for adsorbing heavy metals, which was usually within 2 to 6 h^30–32^.

**Figure 3:**
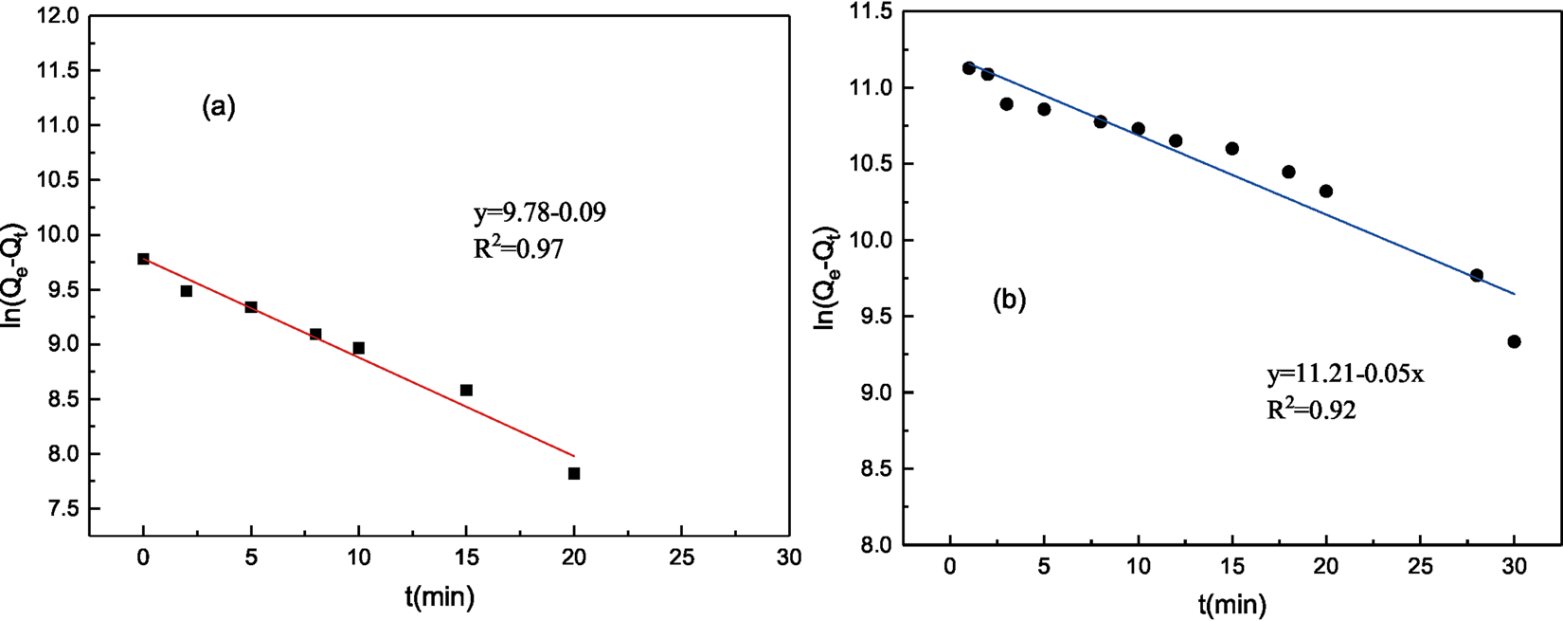
The simulated results of pseudo-first order kinetics on C_16_TAB (**a**) and tea saponin (**b**).

Additionally, to further evaluate the adsorption kinetics and mechanism, pseudo-first order kinetic model was employed to interpret the kinetic results, as shown in Fig. 3. The coefficients of determination (*R*^2^) values obtained by the pseudo-first order kinetic model were 0.97 and 0.92 for C_16_TAB and tea saponin, respectively. Besides, the calculated *Q*_e_ values were in good agreement with the experimental results, proving that the pseudo-first order kinetic model was an appropriate approach to describe the adsorption. This fitting model is consistent with that of *C. vulgaris* adsorbing most heavy metals like Cu^2+^ and Cd(II)^33,34^. Kinetic data of the two surfactants onto *C. vulgaris* implied that the adsorption was dominated by physical forces. Noticeably, the adsorption quantity of C_16_TAB was higher than that of tea saponin in the present experimental conditions, as there were more adsorption sites for C_16_TAB than that for tea saponin on the cell wall^35^. In addition, the combination of the algae and the C_16_TAB was deduced to be a coordination or complexation formation, due to the help of alginate and sulfated polysaccharides of algae.

### Adsorption isotherm

**Figure 4:**
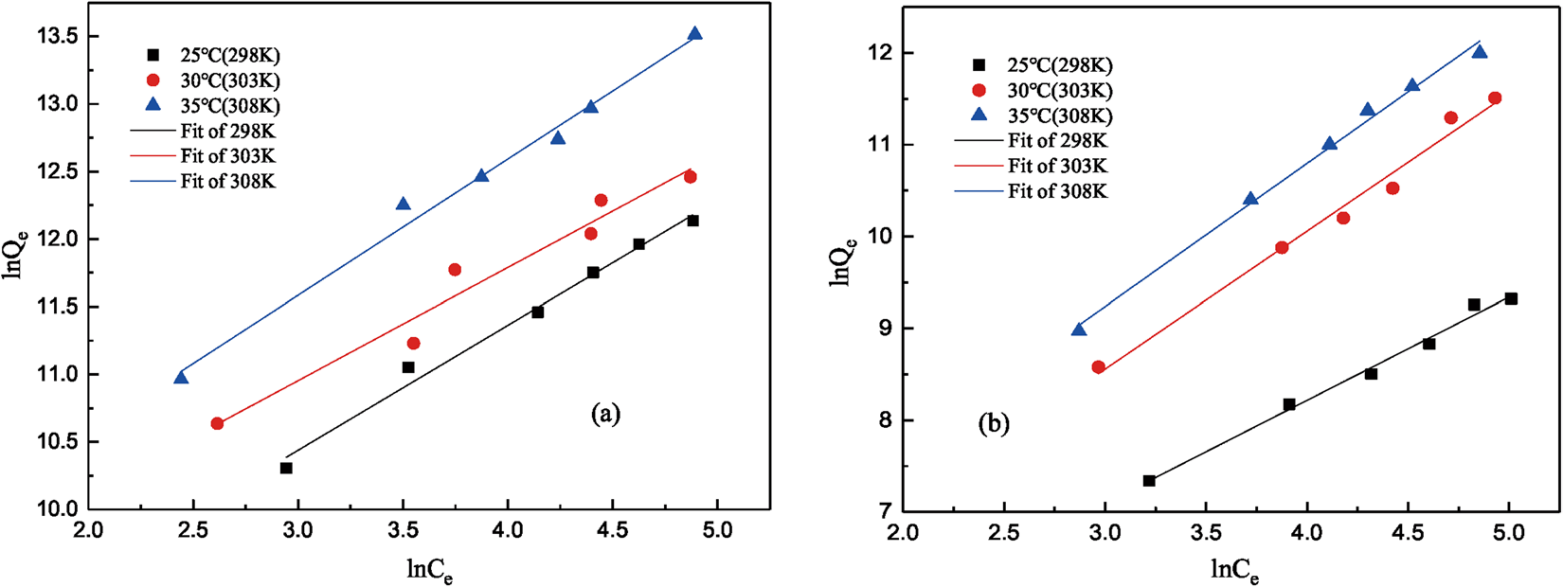
Experimental data and fitting in the Freundlich model of C_16_TAB (**a**) and tea saponin (**b**) under different temperatures (298 K, 303 K, 308 K).

The adsorption isotherms of C_16_TAB and tea saponin onto *C. vulgaris* were performed at 25 °C (298 K) with the solution pH close to 7.0^36^. In the adsorption isotherm experiments the concentrations of both surfactants used were 25 mg/L, 50 mg/L, 75 mg/L, 100 mg/L, 125 mg/L and 150 mg/L, respectively. The equilibrium time of C_16_TAB and tea saponin was 20 and 25 min respectively, which had been demonstrated in the adsorption kinetics. Figure 4 showed the relationship between adsorption capacities of these surfactants onto *C. vulgaris* under different temperatures. From these results it could be observed that the adsorption capacity increased with an increase in the concentration of C_16_TAB, and a similar trend has been observed for tea saponin. Based on the adsorption data obtained from experiments, the sorption thermodynamics was fitted into Freundlich isotherm model^37^. The linearized form of Freundlich isotherm model can be written as follows:

4 $${ln}\,{Q}_{e}=\,{ln}\,{K}_{F}+\frac{1}{n}\,{ln}\,{C}_{e}$$

where *Q*_*e*_ (mg/kg) and *C*_*e*_ (mg/L) are the sorption uptake and concentration of surfactants at equilibrium, respectively. *K*_*F*_ ((mg/kg)·(L/mg)^1/n^) is a constant for relative adsorption capacity, and *n* is the heterogeneity factor. The values of *K*_*F*_ and *1/*n can be calculated from the intercept and from the slope of the linearized model of Freundlich isotherm. The values of coefficient of determination (*R*^2^) for both C_16_TAB (*R*^2^ = 0.986) and tea saponin (*R*^2^ = 0.989) were close to 1, suggesting that the model can provide satisfied fitting features. Furthermore, the residual sum of squares was small, 0.026 for C_16_TAB and 0.025 for tea saponin, respectively, which demonstrated that the isotherm process was suitable for the Freundlich model.

As for the Freundlich isotherm model, the adsorption is considered to be an easy process when *1/n* value is in between 0.1 and 1. However, desorption and regeneration would be feasible at higher values. Moreover, *1/n* for C_16_TAB was lower than 1, indicating that algae adsorbed by C_16_TAB was easier than tea saponin (*1/n* = 1.220). Meanwhile, higher value of *K*_*F*_ indicates the high adsorption capacity of surfactants for the algae^38^. Since *K*_*F*_ of C_16_TAB was much larger than tea saponin, it could be inferred that a larger quantity of C_16_TAB was adsorbed onto *C. vulgaris* than tea saponin.

Unlike the common isotherm models such as Langmuir, Freundlich isotherm is widely used to describe the adsorption onto heterogeneous surfaces where multilayer sorption occurs on the surfaces^39^. It differed with the results obtained from the kinetics model, as pseudo-first order model was assumed to describe a direct physical adsorption process. The differences observed may be due to different sorption sites. The biosorption first occurred on the cell wall, which was composed of 24–74% polysaccharide, 2–16% protein and 1–24% uronic acid. These components offer several functional groups for the adsorption, such as amino, acylamino, carbonyl, aldehyde and hydroxyl^35^, which play a pivotal role in binding with organic groups. Subsequently, the above organisms would pass through the cell wall and enter into the cytomembrane which is permselective. These will lead the algae to enrich the organism. These processes were generally named as biosorption. Yang *et al*.^40^ studied the biosorption of nickel on *Sargassum sp*, one strain of brown algae, and obtained a similar result that the isotherm matched the Freundlich model successfully.

### Thermodynamics

Thermodynamic parameters, Δ*G*, Δ*H* and Δ*S*, were calculated for assessing the biosorption. As the experimental data were described in the Freundlich model, the value of *K*_*F*_ at different temperatures (398 K, 303 K, 308 K) was used to calculate the above parameters through the following equations:

5 $${\rm{\Delta }}G=-RT\,{ln}\,{K}_{F}$$

6 $${\rm{\Delta }}G={\rm{\Delta }}H-T{\rm{\Delta }}S$$

where *K*_*F*_ is the Freundlich constant, *R* is the gas constant which equals to 8.314 J(mol·K)^−1^, and *T* is the thermodynamic temperature in K. The obtained values of thermodynamic parameters are given in Table 1.

**Table 1:** Thermodynamic parameters for C_16_TAB and tea saponin adsorption onto *C. vulgaris*.

	Δ*H* (kJ/mol)	Δ*S* (kJ/mol)	Δ*G* (kJ/mol)		
			298 K	303 K	308 K
C_16_TAB	70.659	0.314	−22.806	−24.374	−26.784
Tea saponin	15.987	0.121	−20.112	−20.863	−21.325

As shown in Fig. 4, the adsorption capacity of these two surfactants onto *C. vulgaris* increased with an increase in temperature, which demonstrated that the adsorption was favored by higher temperature and further indicated that it might be an endothermic process. Comparing with the values of *Q*_*e*_, C_16_TAB showed a considerably higher value than tea saponin, which also proved its better adsorption. For both surfactants, *C. vulgaris* adsorption amount was more at high concentration and at high temperatures. This was clearly supported by the positive values of Δ*H*, indicating an endothermic process^41^. Therefore, the dosage of surfactant adsorbed onto algae increased with an increase of temperature. Δ*S* indicates the randomness in the process; positive values of both C_16_TAB and tea saponin indicated that after adsorption, there might be some changes occurring on the structure at the algae/dosage interface^42^, resulting in an increase in the randomness. In addition, if the value of Δ*G* is in the range between 0 and 20 kJ/mol, the adsorption is considered as physisorption, whereas if the value of Δ*G* ranges from −400 to −80 kJ/mol, the adsorption can be recognized as chemisorption^43^. For the *C. vulgaris*, the present value of *ΔG* was in the range of −20.112~−26.784 kJ/mol, indicating that the present adsorption was neither physisorption nor chemisorption. Besides, this was also found in plastic flotation^44^. This adsorption was probably due to the hydrogen bond. Hydrogen bond is an interaction stronger than Van der Waals’ force but weaker than chemical bond^45^. It comes from the electrostatic force between the hydrogen core in strong polar bond and atom with negative charge, like X-H…Y, where X and Y are on behalf of some negative non-metallic element such as O and N^46^. There are lots of hydroxy on the algal surface^13^. On the other hand, C element could be regard as electron acceptor. Therefore it could be the end of the hydrogen bond^47^, and it also could be found on *C. vulgaris*.

### Interaction mechanisms between algae and surfactants

**Figure 5:**
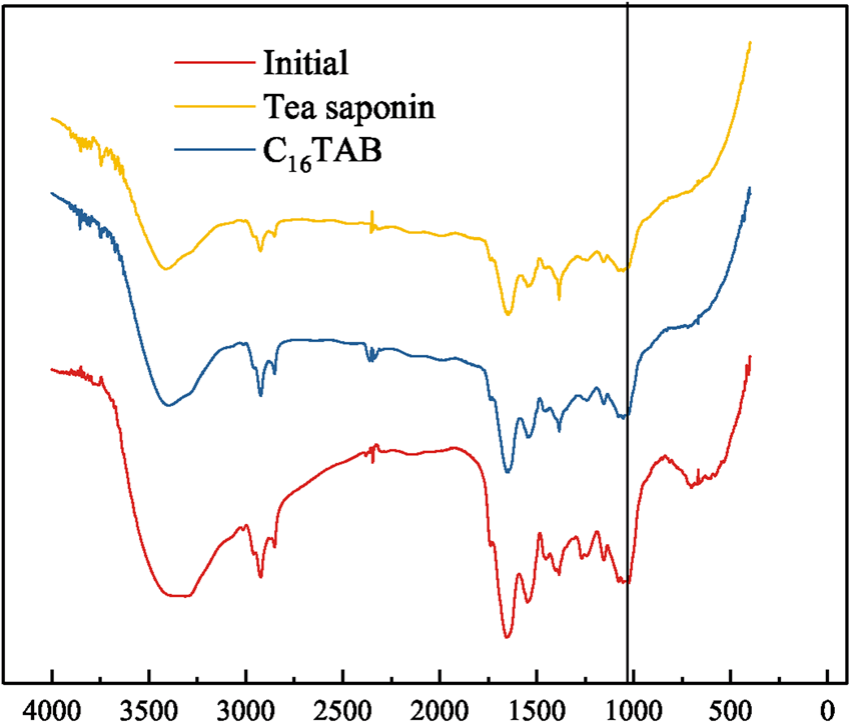
FT-IR spectra of initial *C. vulgaris* and after treatment of C_16_TAB, tea saponin.

In order to elucidate the mechanism of adsorption with the two surfactants and to ascertain whether the chemisorption occurred on the surface of microalgae, FT-IR was utilized in this study. FT-IR spectra of *C. vulgaris* treated and untreated with surfactants under an optimal dosage were recorded in Fig. 5. Green algae mainly have cellulose in the cell wall, and a high content of proteins is bonded to the polysaccharides^48^. It can be observed in Fig. 5 that C-H stretching mode can be observed at 2924 cm^−1^. Besides, NH_2_ stretching mode at 1548 cm^−1^, -SO- stretching mode at 1400 cm^−1^, -C = O molecular vibrations at 1048 cm^−1^ and at 1655 cm^−1^ could be seen. No obvious change in the FT-IR spectrum between *C. vulgaris* untreated and treated by the C_16_TAB was observed, which indicated that no new chemical groups were introduced on the surface of *C. vulgaris*. Nevertheless, after treated by the tea saponin, there was change happened at 1048 cm^−1^. It can be seen that there was a peak after treated, which might be due to the -COOH reaction with -OH or -NH2^49^, from the hydrophilic part of tea saponin and the surface of *C.vulgaaris* respectively. Moreover, this reaction was supposed to be reversible with low equilibrium constant, and the Δ*G* of tea saponin might be explained in this way.

### Adsorption mechanism of surfactants

According to the results of adsorption experiments and FT-IR measurements, the adsorption of C_16_TAB was neither a chemisorption process nor a physisorption process. As discussed before, the addition of C_16_TAB caused an increase in the algal potential obviously due to their charge reversal. Therefore, the electrostatic forces played an important role in the adsorption. Moreover, because bubbles also carried the negative charge, the algae were prone to connecting bubbles with the C_16_TAB by neutralization. On the other hand, there should be an interaction stronger than simple physical behavior, as the Δ*G* was higher than 20 kJ/mol, but the FT-IR spectra didn’t show any sign of chemical interaction. The protein could form hydrogen bonds with oppositely charged ions^50^. It should be noticed that there were lots of protein and phospholipid layer in the algal surface^51^. Therefore, the protein on the surface of *C. vulgaris* might form the hydrogen bonds with C_16_TAB, and this bonds fitted the condition well in which it was not a chemical interaction but stronger than simple physical behavior.

**Figure 6:**
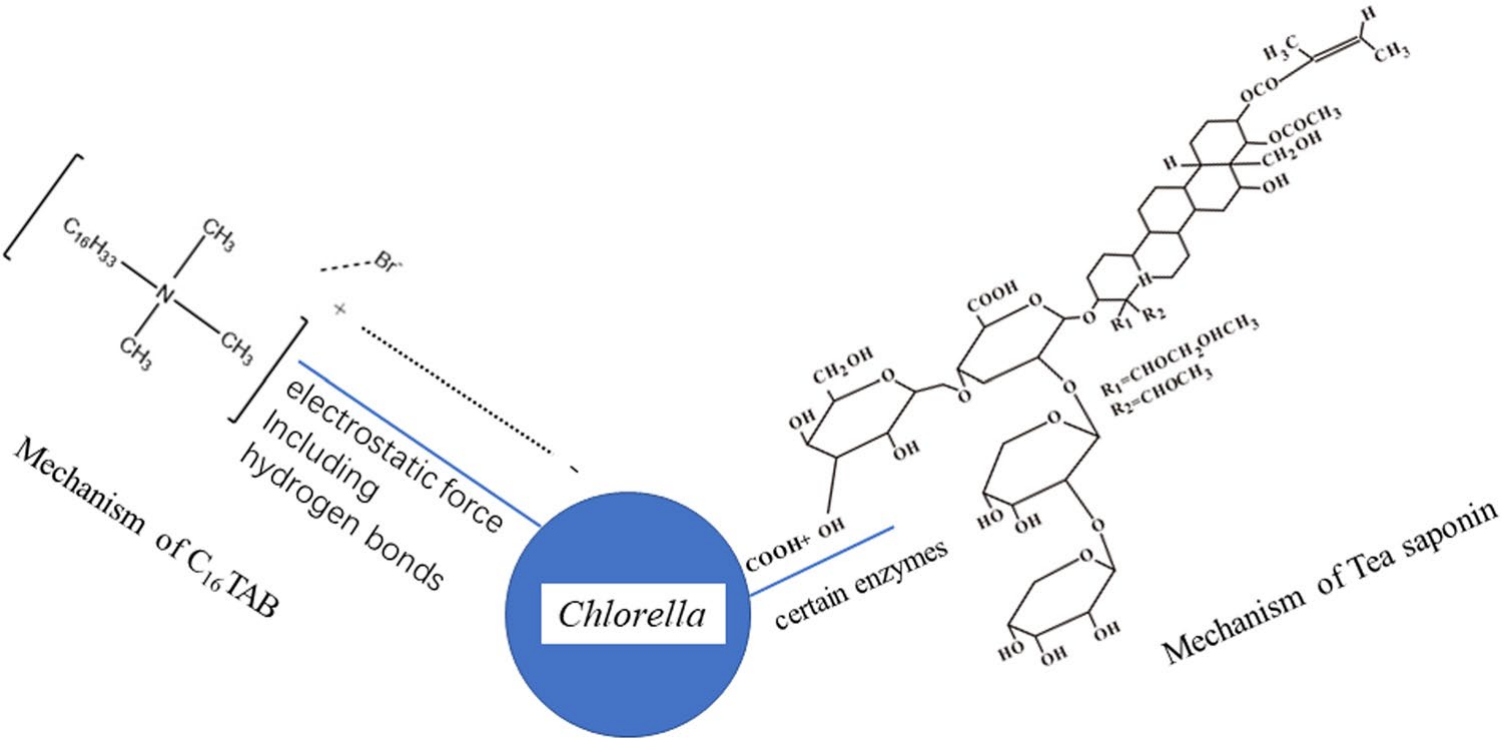
Adsorption of C_16_TAB and Tea saponin onto the *C. vulgaris*.

It could be concluded that tea saponin adsorption was a physisorption in general, accompanied with a reversible interaction between carboxyl and hydroxyl polyreaction. Tea saponin was a neutral surfactant so that the electrostatic forces cannot play a leading role in the adsorption. However, it contained a large amount of hydroxyl groups, which could act as an important role in the polyreaction. Based on the FT-IR results discussed in 3.5, this might be attributed to those carboxyl groups in the proteins that were on the surface of *C. vulgaris*. The carboxyl groups would react with the hydroxyls or amidogens involved in tea saponin, which was promoted by certain enzymes^49^. Furthermore, tea saponin was a biological product and had a good biocompatibility which might make this reaction possible. In summary, the adsorption mechanisms of C_16_TAB and tea saponin on to *C. vulgaris* were shown in the Fig. 6.

**Figure 7:**
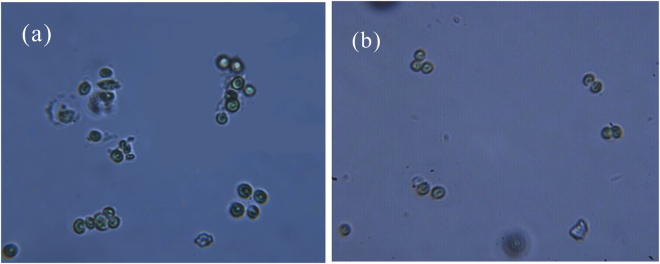
Microscopic images of flocs in the surfactant solutions of C_16_TAB (**a**) and tea saponin (**b**) (amplification 1:1000).

It is well known that surfactants could not only provide foams, but also change some surface properties such as hydrophobicity and Zeta potential^52^. Surfactants would lower the repellency between algal cells and cells. This can promote the formation of flocs, which would improve significantly harvesting performance. Figure 7 showed the microscopic images of flocs in the surfactant solutions of C_16_TAB and tea saponin. It can be seen that the flocs treated by C_16_TAB consisted of multiple cells, while the flocs treated by tea saponin contained just two or three cells. These phenomena could demonstrate the existence of the electrostatic neutralization between C_16_TAB and algae to some extent. Further this force could have impact on most of the cells, resulting in decreasing the energy requirement for the cells to flocculate with each other. However, the reaction caused by tea saponin, which was between the carboxyl and hydroxyl, was limited by certain factors and can only have impact on a few algae. Therefore, this was one of reasons why the flotation efficiency of *C. vulgaris* with the addition of C_16_TAB was better than tea saponin.

Table 2 summarized the contact angles measured with the examined liquids and the physico-chemical properties of microalgae. Surface hydrophobicity of the material can be characterized by the contact angle. The free energy of cohesion (*ΔG*_*coh*_) represented the hydrophobicity of material. In general, the positive value of *ΔG*_*coh*_ means hydrophilic surface and the negative value suggests hydrophobic. It could be found that *C. vulgaris* was a hydrophilic strain of algae in nature (*ΔG*_*coh*_=1.21), and the addition of tea saponin would change the surface feature of *C. vulgaris* to hydrophobic owing to a negative value of *G*_*coh*_. However, in the presence of C_16_TAB the situation was completely different. Almost no contact angle could be measured in all the three tested liquids. Therefore, their hydrophobicity could not be determined, and such phenomenon was referred as spreading wetting^53^. As results, the hydrophilic part of C_16_TAB should be outside of the cell, while the hydrophobic tip may be outside when tea saponin was adsorbed onto the algae. Furthermore, the Freundlich model had revealed that there were multilayers of surfactants adsorbed on the surface. Combining with the results obtained from contact angles, it could be noted that C_16_TAB adsorbed onto the algae cells should be evenly layered while tea saponin should be odd layered.

**Table 2:** The surface characteristics of *C. vulgaris* before and after treated by surfactant.

	_Contact angles/(°)_			Δ*G*_coh_
	*θ*_water_	*θ*_*glycol*_	*θ*_*glycerol*_	
*C. vulgaris*	49 ± 0.9	12 ± 0.43	45 ± 1.1	1.21
C_16_TAB	0	0	0	—
Tea saponin	31 ± 0.245	19 ± 0.479	48 ± 0.197	−8.61

**Figure 8:**
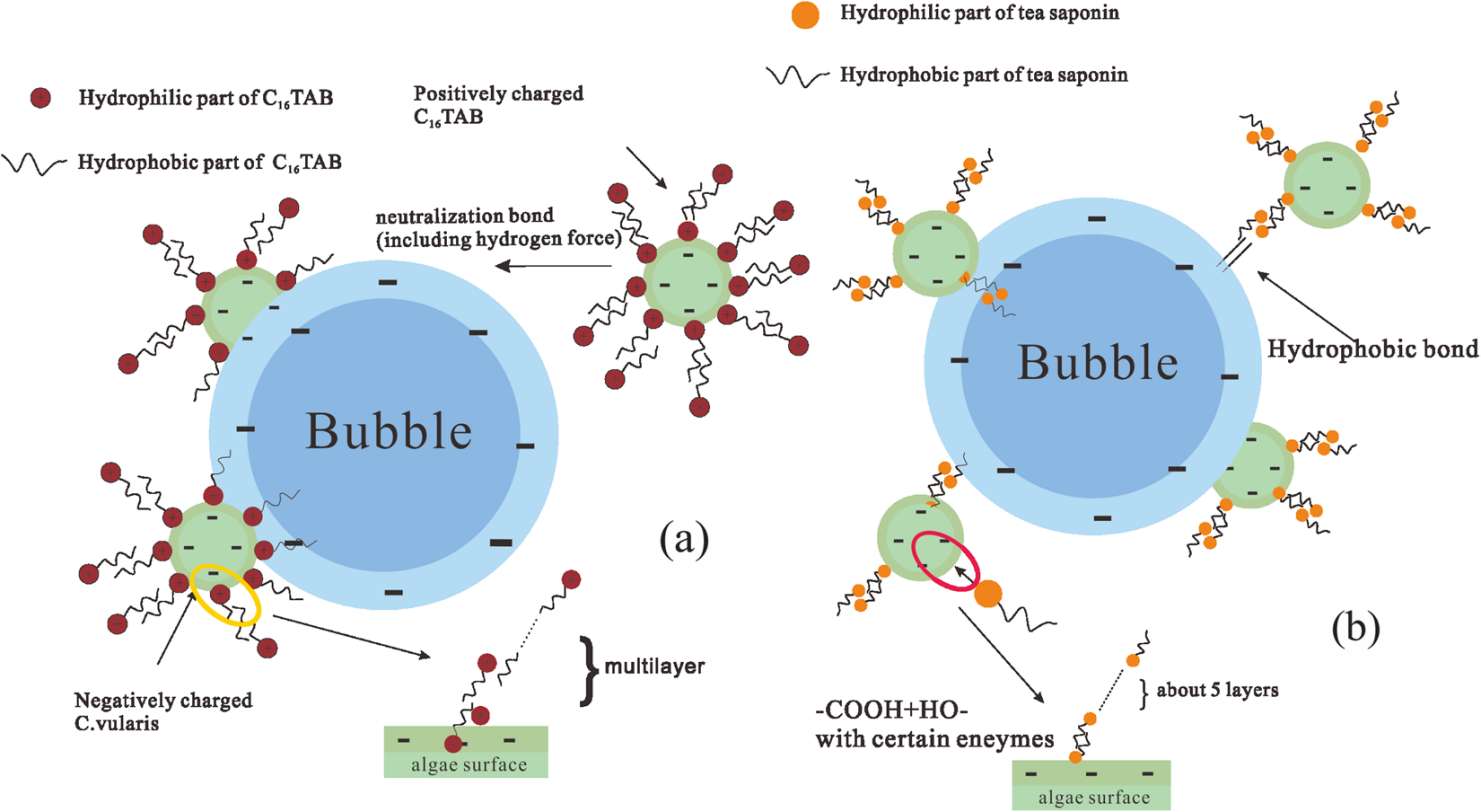
Mechanism models for the interactions among algae, surfactants and bubbles in the C_16_TAB-aided (**a**) and tea saponin–aided (**b**) flotation.

In order to deeply understand how surfactants influence the attachment performance in flotation process, two mechanism models for the interactions among algae, surfactants and bubbles could be proposed as shown in the Fig. 8. With regard to C_16_TAB-aided flotation, the surfactant C_16_TAB is a cationic surfactant, which can connect *C. vulgaris* by electric neutralization reaction^41^, and form hydrogen bond as mentioned previously. Meanwhile, some parts of C_16_TAB are hydrophilic and the others are hydrophobic^54^. Therefore, the hydrophilic parts were attached to *C. vulgaris* as it was hydrophilic^55^, which maybe another possible reason for the adsorption. Afterwards, this complex containing algae and C_16_TAB was more positive and hydrophobic outside. It was recognized that bubbles in the flotation are electronegative^29^ and hydrophobic. Therefore, this complex could attach to the bubble easily, resulting in a higher flotation recovery.

The mechanism in tea saponin-aided flotation was not similar to that of C_16_TAB-aided flotation. The tea saponin influenced the algae by the hydrophobic attraction and van der Waals force including a portion of polymerization reaction between the hydroxyl and carboxyl groups with certain enzymes^49^, whereas its equilibrium constant was too low to be stable. This might be the reason for the Δ*G* in the range of −20 kJ/mol and −80 kJ/mol, resulting in a lower adsorption and recovery than C_16_TAB. As a kind of biosurfactant, tea saponin had both hydrophilic and hydrophobic parts, which could help to enhance the bond between algae and bubble as a bridge.

## Conclusions

To deeply understand the role of surfactants in bubble-algae attachment interaction, two mechanism models were established to reveal the relationship among the algae, surfactants and flotation performance based on the results of adsorption of C_16_TAB and tea saponin onto *C. vulgaris*. Surfactants did play an important role in flotation process: C_16_TAB exhibited better collecting performance than tea saponin and the harvesting efficiency reached 89.23% at C_16_TAB concentration of 80 mg/L. Compared to tea saponin, C_16_TAB could adsorb onto *C. vulgaris* more easily and the interaction between them was more stable. Further FT-IR analysis confirmed that no new chemical groups were introduced on the surface of *C. vulgaris* after being treated by C_16_TAB, while the interaction about hydroxyl and carboxyl was verified after treated with tea saponin. It could be speculated that the neutralization and hydrophobic bond should be the major reason for the better adsorption. However, in this study, the *C. vulgaris* was supposed to produce the biofuel, thus the concentration of C_16_TAB was not a serious problem. If the algae was planned to be drag or fodder, the toxicity and concentration of surfactants need to be further considered. In the future work, more variety of surfactants and algae should be examined to obtain some common indexes, which may be helpful to identify which surfactant is more effective and appropriate for achieving higher flotation recovery.
